# Phylogeny of anaerobic fungi (phylum *Neocallimastigomycota*), with contributions from yak in China

**DOI:** 10.1007/s10482-016-0779-1

**Published:** 2016-10-12

**Authors:** Xuewei Wang, Xingzhong Liu, Johannes Z. Groenewald

**Affiliations:** 1 State Key Laboratory of Mycology, Institute of Microbiology, Chinese Academy of Sciences, No. 3, 1st Beichen West Road, Chaoyang District, Beijing, 100101 China; 2 CBS-KNAW Fungal Biodiversity Centre, Uppsalalaan 8, 3584 CT Utrecht, The Netherlands

**Keywords:** Anaerobic fungi, Morphology, Phylogenetic relationships, Yak

## Abstract

The phylum *Neocallimastigomycota* contains eight genera (about 20 species) of strictly anaerobic fungi. The evolutionary relationships of these genera are uncertain due to insufficient sequence data to infer their phylogenies. Based on morphology and molecular phylogeny, thirteen isolates obtained from yak faeces and rumen digesta in China were assigned to *Neocallimastix frontalis* (nine isolates), *Orpinomyces joyonii* (two isolates) and *Caecomyces* sp. (two isolates), respectively. The phylogenetic relationships of the eight genera were evaluated using complete ITS and partial LSU sequences, compared to the ITS1 region which has been widely used in this phylum in the past. Five monophyletic lineages corresponding to six of the eight genera were statistically supported. Isolates of *Caecomyces* and *Cyllamyces* were present in a single lineage and could not be separated properly. Members of *Neocallimastigomycota* with uniflagellate zoospores represented by *Piromyces* were polyphyletic. The *Piromyces*-like genus *Oontomyces* was consistently closely related to the traditional *Anaeromyces*, and separated the latter genus into two clades. The phylogenetic position of the *Piromyces*-like genus *Buwchfawromyces* remained unresolved. *Orpinomyces* and *Neocallimastix*, sharing polyflagellate zoospores, were supported as sister genera in the LSU phylogeny. Apparently ITS, specifically ITS1 alone, is not a good marker to resolve the generic affinities of the studied fungi. The LSU sequences are easier to align and appear to work well to resolve generic relationships. This study provides a comparative phylogenetic revision of *Neocallimastigomycota* isolates known from culture and sequence data.

**Electronic supplementary material:**

The online version of this article (doi:10.1007/s10482-016-0779-1) contains supplementary material, which is available to authorized users.

## Introduction

Since their first recognition as Fungi in the mid-1970s (Orpin 1975), the anaerobic fungi have been widely recognised as active and major contributors to the degradation of plant fibre within the rumen and hindgut of larger herbivorous animals (Bauchop 1981; Wood et al. 1986; Joblin et al. 1989; Trinci et al. 1994). They are not only crucially involved in the rumen function and animal nutrition, but also have great potential for improving the conversion of lignocellulose into bioenergy products (Dashtban et al. 2009; Youssef et al. 2013; Haitjema et al. 2014; Solomon et al. 2016). These organisms were initially described only as a group of chytridiomycetous fungi (Orpin 1975) due to the attributes of their life cycle (with a vegetative structure from which zoospores are produced) and biochemistry (chitin-containing cell walls). Later, based on the type species *Neocallimastix frontalis*, Heath et al. (1983) formally classified them into a new family *Neocallimastigaceae* in the chytridiomycetous order *Spizellomycetales* mainly due to the similarities of zoospore ultrastructure to some members of this order. Their taxonomic position in *Spizellomycetales* was then contended by molecular data (Li and Heath 1992), and the order *Neocallimastigales* was proposed for the family based on the comparison of multiple morphological, ultrastructural, and developmental characters among the *Chytridiomycota* (Li et al. 1993). Further phylogenetic analyses with nrDNA (18S + 5.8S + 28S) from an extensive range of chytridiomycetous fungi revealed *Neocallimastigales* to be a monophyletic group within the polyphyletic *Chytridiomycota* (James et al. 2006). Combined with morphological, ecological and ultrastructural data, the phylum *Neocallimastigomycota* was subsequently erected (Hibbett et al. 2007).

On the basis of the thallus morphology and the number of flagella per zoospore, six genera were described (Ho and Barr 1995; Ozkose et al. 2001; Gruninger et al. 2014). There are four genera possessing rhizoidal thalli composed of a branched rhizoidal system and sporangia: *Anaeromyces* has polycentric thalli (exhibiting multiple centers of reproduction with more than one sporangia in common) and uni-flagellate zoospores; *Neocallimastix* possesses monocentric thalli (with one reproductive body or sporangium) and multi-flagellate zoospores; *Orpinomyces* has polycentric thalli and multi-flagellate zoospores; *Piromyces* possesses monocentric thalli and uni-flagellate zoospores. Two other genera, *Caecomyces* and *Cyllamyces*, produce thalli composed of bulbous vegetative cell (or holdfast) and sporangia. *Caecomyces* normally possesses only one, or a limited number of, zoosporangia formed directly on the surface of the bulbous holdfast or on the end of a simple sporangiophore (Wubah et al. 1991a; Ho and Barr 1995; Fig. 1 in Gruninger et al. 2014); while multiple zoosporangia are produced on branched sporangiophores with nuclei for *Cyllamyces* (Ozkose et al. 2001). In addition, uni-flagellate zoospores are defined by predominantly one flagellum per spore, with up to 10 % of zoospores having 2–4 flagella; while multi-flagellate zoospores are characterised by more than four flagella per spore.

**Fig. 1:**
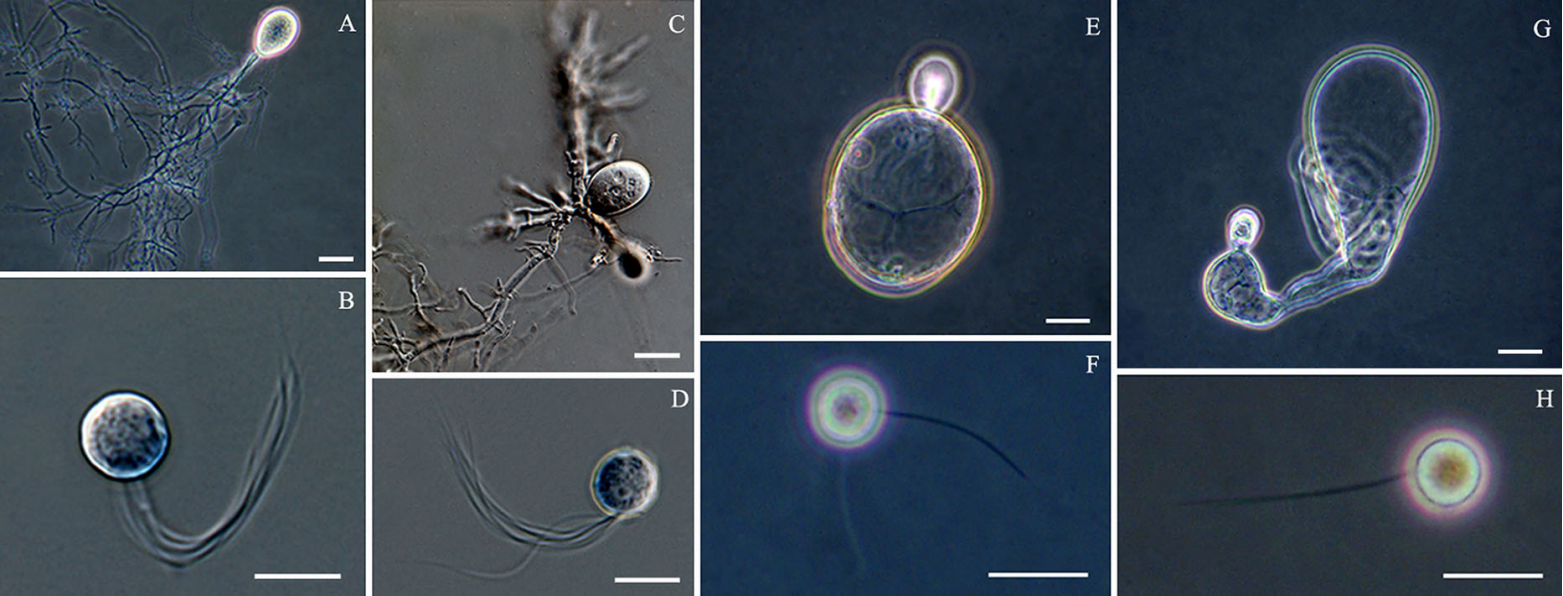
Morphology of anaerobic fungi from yak. *Neocallimastix* sp.: **a** thallus, **b** zoospore; *Orpinomyces* sp.: **c** thallus, **d** zoospore; *Caecomyces* sp.: **e**, **g** thalli, **f**, **h** zoospores. (*bars* **a**, **c**, **e**, **g** = 20 µm; **b**, **d**, **f**, **h** = 10 µm)

From the 1970s to 1990s, nearly 20 species of anaerobic fungi were defined primarily by zoospore ultrastructure (Heath et al. 1983; Orpin and Munn 1986; Munn et al. 1988; Webb and Theodorou 1991), including four species in the genus *Neocallimastix*, three species in the genus *Orpinomyces* and two species in the genus *Caecomyces*. However these ultrastructural criteria have been questioned, not only due to the complicated techniques used for ultrastructural study, but also to the fact that these features tend to vary with culture conditions, age, as well as technique used for sample preparation (Ho and Barr 1995; Brookman et al. 2000).

Several studies attempted to delimit the species morphology under the light microscope. Wubah et al. (1991b) compared two cultures of *N. frontalis* from New Zealand with a culture of *Neocallimastix patriciarum* from the UK. They then concluded that these cultures belonged to the same species. In 1995, a monographic study by Ho and Barr critically examined the thallus morphology of all available species using a light microscope. A broad range of morphological variation of a single species was frequently observed. It was also found that media, stage of growth, etc. could easily influence thallus shape and size. For example, when the medium is rich in glucose or contains filter paper, the sporangia often became abnormally large and abort. Fourteen species were re-described by Ho and Barr (1995). In the genus *Neocallimastix*, the type species *N. frontalis* was observed to produce endogenous or exogenous sporangia quite variable in shape (spherical, ellipsoidal, broad ellipsoidal, ovoid, broadly ovoid or pyriform, occasionally angular, tubular or irregular) and in size (8.5–170 µm diam, or 10 to over 100 µm long), zoospores were also variable in shape (ovoid to globose) and size (7–22 µm diam.). Based on their morphological investigation, they treated both *N. patriciarum* and *N. variabilis* as synonyms of *N. frontalis* because both of them fell within the morphological limits of *N. frontalis*. The status of the third species, *Neocallimastix hurleyensis*, as a separate species was also questioned. *N. hurleyensis* was originally reported to have minor ultrastructural differences from *N. frontalis* (Webb and Theodorou 1988), and then was considered to be different in having zoospores discharging through a clearly defined apical pore in the sporangium from those of *N. frontalis* which released through an apical pore accompanied by dissolution and rupture of the sporangium wall (Webb and Theodorou 1991). But Wubah et al. (1991b) did not accept zoospore discharge as an unequivocal difference between *N. hurleyensis* and *N. frontalis*. In the genus *Orpinomyces*, *O. joyonii* and *O. intercalaris* were recognised, and *O. bovis* was reduced to a synonym of *O. joyonii*. *Orpinomyces joyonii* (originally described by Li et al. 1991) was distinguished by the terminal formation of sporangia on the simple or branched sporangiophores, while *O. intercalaris* (described by Ho et al. 1994) was characterised by sporangia developing intercalarily from expansion of hyphae or as a lateral outgrowth of a hypha, rarely terminal. In the genus *Caecomyces*, *C. equi* was described, on the basis of a cultured material embedded in plastic, as monocentric thallum producing a sporangium on a single bulbous vegetative cell with attached fibrillar or coralloid rhizoids; and *C. communis* was described as one, two or more sporangia on single or multiple bulbous vegetative cells (Ho and Barr 1995). The authors found that, in culture, the single sporangium form of *C. communis* predominated in the first day of culture, and that thalli with several bulbous vegetative cells and two or more sporangia occurred frequently in older cultures. Consequently, the status of *C. equi* as a distinct species was questioned (Ho and Barr 1995). Since no culture of *C. equi* was available for further study, it is difficult to determine whether the two species are synonymous or not. Additionally, six *Piromyces* species and two *Anaeromyces* species were recognised by Ho and Barr (1995). Apparently, the delimitation of some species remained contentious, which gave rise to the publication of a large number of hitherto-unnamed isolates (Brookman et al. 2000; Fliegerová et al. 2004; Sirohi et al. 2013a; Youssef et al. 2013).

Li and Heath (1992) made the first attempt to reveal the relationships among the anaerobic fungi using DNA sequence data. Based on the less conserved ITS1 (first internal transcribed spacer region of the nrDNA cistron) sequence data from single *Anaeromyces*, *Neocallimastix*, *Orpinomyces* and *Piromyces* isolates, the authors showed *Anaeromyces* to be more distant from the other three genera and the relationships of the other genera remained unresolved. Brookman et al. (2000) expanded the ITS1 sequence analysis to include a larger number of isolates and successfully differentiated *Neocallimastix* from *Piromyces*. Their results also revealed the genus *Piromyces* as being divergent. Later, the nrDNA sequences from the type material of the *Cyllamyces* type species (*Cyllamyces aberensis*) were made publicly available (James et al. 2006), and the D1/D2 domain of the 28S nrDNA gene (LSU) was used to differentiate the species of *Orpinomyces* (Dagar et al. 2011). Using molecular data, two more genera, *Oontomyces* (Dagar et al. 2015) and *Buwchfawromyces* (Callaghan et al. 2015) were proposed recently. Both genera morphologically fit with the genus *Piromyces*, but genetically appeared much closer to the genus *Anaeromyces*. However, the evolutionary relationships of different genera remain open for discussion, and the phylogenetic affiliations of species were challenged more frequently not only because the highly variable nature of the ITS region in this phylum makes it difficult to get a reliable phylogenetic tree, but also because most described species of anaerobic fungi lack sequence data from their type specimens (Griffith et al. 2010; Gruninger et al. 2014). On the other hand, ITS1 sequences have been widely employed in the studies of both diversity and ecology of the anaerobic fungi in the environment (Griffith et al. 2009; Nicholson et al. 2010; Fliegerová et al. 2010; Liggenstoffer et al. 2010; Kittelmann et al. 2012; Sirohi et al. 2013b; Koetschan et al. 2014). These cultivation-independent investigations have indicated the existence of many putative novel lineages (candidate genera) in this phylum (Nicholson et al. 2010; Liggenstoffer et al. 2010; Gruninger et al. 2014). At the same time, the generic identification of a large number of released ITS1 sequences from anaerobic fungal isolates or clones is doubtful (Nicholson et al. 2010; Fliegerová et al. 2010, Kittelmann et al. 2012). Koetschan et al. (2014) proposed the use of the common core secondary structure of the ITS1 region to reconstruct a more reliable phylogenetic tree of *Neocallimastigomycota*. Over 1100 of complete neocallimastigomycete ITS1 sequences were used, most of which were unclassified and obtained from uncultured samples. The integration of the additional structural information into the phylogenetic analyses allowed the authors to recognise the six known genera, as well as nine other defined monophyletic groups from uncultured sequences waiting for morphological characterisation. In their analysis, ten sequences failed to cluster into any of the defined groups (Fig. 4 in Koetschan et al. 2014); of these, two sequences (GenBank accessions AF170205 and AF170206) were from two isolates morphologically identified as members of *Piromyces* (Brookman et al. 2000), further confirming the polyphyletic nature of the morphologically defined *Piromyces*. Although there has been much progress in both the morphological and phylogenetic study of anaerobic fungi, it is clear that a stable taxonomic backbone complying with the Botanical Code of Nomenclature is urgently required for a better understanding the evolution, ecology and functions of the organisms in the *Neocallimastigomycota* (Ho and Barr 1995; Griffith et al. 2010; Gruninger et al. 2014).

Yak (*Bos grunniens*), as one kind of large ruminants existing on the Qinghai-Tibetan Plateau at elevations of 3000–5500 m, possesses unusual physiological adaptations to the extreme conditions such as low temperature (as low as −40 °C) and full-grazing style with grasses, sedges, and fobs as their sole nutritional source (Wiener et al. 2003; Zhong et al. 2006; Leslie and Schaller 2009). The prokaryotic populations in yak rumen have been reported to be distinct, with more than half of the species belonging to hitherto uncultured groups of prokaryotes (An et al. 2005). *Neocallimastix*, *Orpinomyces* and *Piromyces* were reported from yak in recent years (Feng 2005; Wei et al. 2016). However, our understanding of the fungal population diversity inside the yak digestive track is still limited.

In this study, as part of our survey on anaerobic fungi in yak rumen, we isolated fungi from both yak faeces and rumen digesta in Qinghai province, where yaks accounting for nearly half of the total Chinese yak population can be found. The aim of the present study was to examine the diversity of the obtained anaerobic fungal isolates in a phylogenetic context by assessing the phylogenetic relationships among the anaerobic fungi at the genus level based on additional sequences from the GenBank nucleotide database.

## Materials and methods

### Isolation and cultivation

The isolates were obtained from naturally grazing, rumen-fistulated or slaughtered yaks. Two rumen samples were taken directly from an animal carcas at local yak slaughterhouses in Qinghai province, China: one in Jiegu county of Yushu, and the other in Xining city. Five other rumen samples were taken at different time points from the two nonlactating rumen-fistulated yaks housed at the Academy of Animal Science and Veterinary Medicine affiliated to Qinghai University in Xining, China. All the rumen samples were promptly put into anaerobic Hungate tubes containing 10 ml modified Orpin’s medium C (Theodorou et al. 2005), and then used directly for purification in order to get the maximum coverage of the fungal diversity in the rumen using the rolling tube method described below. All animal procedures were approved by the Committee on the Ethics of Animal Experiments of the Institute of Microbiology, Chinese Academy of Sciences, China (permit number: PZIMCAS2008001) and every effort was devoted to minimising suffering of the rumen-fistulated yaks. For the naturally grazing yaks, fresh faeces were collected from the pastures in Qinghai province. The faecal samples were air-dried for transportation and were used for isolation within 1 week after collection. Isolation and four cycles of purification by the rolling tube method were performed as described by Theodorou et al. (2005). All the isolates were maintained in modified Orpin’s medium C with 8 g milled wheat straw l^−1^ as carbon source. Detailed information about all novel isolates are listed in Table 1. The morphological features of thalli and zoospores were examined by phase contrast observation using a Zeiss Axioskop 2 microscope or by DIC observation using a Nikon ECLIPSE 80i microscope.

**Table 1:** Collection details and GenBank accession numbers of the isolates obtained in this study

Taxon	Strain	Source	GenBank accession numbers	
			ITS1/ITS	LSU
*Caecomyces* sp.	CYF	Yak faeces, Guoluo, Qinghai Province, China	JQ782554	JQ782554
*Caecomyces* sp.	CYR	Rumen of rumen-fistulated Yak A, Xining, Qinghai Province, China	JQ782555	JQ782555
*Neocallimastix frontalis*	NYF1	Yak faeces, Guoluo, Qinghai Province, China	JQ782542	JQ782542
*Neocallimastix frontalis*	NYF2	Yak faeces, Yushu, Qinghai Province, China	JQ782543	JQ782543
*Neocallimastix frontalis*	NYF3	Yak faeces, Guoluo, Qinghai Province, China	JQ782544	JQ782544
*Neocallimastix frontalis*	NYF4	Yak faeces, Hainan, Qinghai Province, China	JQ782545	JQ782545
*Neocallimastix frontalis*	NYR1	Rumen of rumen-fistulated Yak A, Xining, Qinghai Province, China	JQ782546	JQ782546
*Neocallimastix frontalis*	NYR2	Rumen of rumen-fistulated Yak A, Xining, Qinghai Province, China	JQ782547	JQ782547
*Neocallimastix frontalis*	NYR3	Rumen of rumen-fistulated Yak B, Xining, Qinghai Province, China	JQ782548	JQ782548
*Neocallimastix frontalis*	NYR4	Rumen of rumen-fistulated Yak B, Xining, Qinghai Province, China	JQ782549	JQ782549
*Neocallimastix frontalis*	NYR5	Yak rumen, Xining, Qinghai Province, China	JQ782550	JQ782550
*Orpinomyces joyonii*	OYF	Yak faeces, Hainan, Qinghai Province, China	JQ782551	JQ782551
*Orpinomyces joyonii*	OYR2	Yak rumen, Yushu, Qinghai Province, China	JQ782553	JQ782553

### DNA extraction and PCR amplification

Total genomic DNA was extracted from cultures using the E.Z.N.A.™ High Performance (HP) Fungal DNA Kit (Omega Bio-tek, USA) following the manufacturer’s instructions. The complete ITS1/5.8S nrRNA gene/ITS2 regions (ITS) and the D1/D2 domains of 28S nrDNA (LSU) were amplified in a Hybaid Px2 Thermal Cycler (Thermo Scientific, USA) using the primer combination ITS5 (White et al. 1990) and NL4 (O’Donnell 1993). The PCR reaction (50 μl total volume) contained 0.25 μM of each primer, 0.5 mM dNTP, 0.8 U of *Taq* DNA polymerase in 1× reaction buffer containing 2 mM MgCl_2_ (Transgen, China), and with 2 μL of template gDNA. The PCR program consisted of an initial denaturation of 5 min at 95 °C followed by 30 cycles of denaturation at 94 °C for 1 min, annealing at 55 °C for 1 min and elongation at 72 °C for 2 min and a final extension of 72 °C for 10 min. After purification using a QIAquick PCR Purification Kit (Qiagen, USA), the PCR products of all strains were cloned into the pGEM®-T Vector System I (Promega, USA). The ligated products were transformed into competent *E. coli* DH5α cells. One of the recombinant colonies of each isolate was randomly selected and sent to SinoGenoMax Co., Ltd. (http://www.sinogenomax.com) for sequencing using a standard M13 primer set in an Applied Biosystems 3730xl DNA Analyzer (Thermo Fisher Scientific, USA).

### Sequence alignment and phylogenetic analyses

The novel sequences obtained for all the isolates were deposited in GenBank under the accession numbers shown in Table 1. The final data sets incorporated all representative sequences publicly available from pure cultures, covering each of the eight known genera (Tables 1, S1). In addition, our sequences were also checked against the expanded dataset of Koetschan et al. (2014) to confirm that none of our sequences fits with any of the uncultured lineages of their phylogeny. As different numbers of reference sequences are available for the ITS1 fragment, the complete ITS region and the partial LSU respectively, the phylogenetic analyses were performed separately using three data sets. Two *Monoblepharella* strains, as representatives of *Monoblepharidales*, were used as outgroup based on its relatively close relationship to anaerobic fungi of *Neocallimastigales* (James et al. 2006). The sequence datasets were initially aligned using MAFFT v. 7 (Katoh and Standley 2013), and were manually optimised using MEGA v. 6 (Tamura et al. 2013). For the ITS data sets, the alignment was obtained with the Q-INS-i method of iterative refinement as implemented in MAFFT to consider their secondary structure due to the highly variable nature of these sequences.

Phylogenetic analyses of individual locus datasets were based on Bayesian inference (BI), Maximum Likelihood (ML) and Maximum Parsimony (MP) analyses. For BI, the best evolutionary model for each partition was determined using MrModeltest2 (Nylander 2004) and incorporated into the analyses. A Markov Chain Monte Carlo (MCMC) algorithm was used to generate phylogenetic trees using MrBayes v. 3.2.1 (Ronquist and Huelsenbeck 2003; Ronquist et al. 2012) under the optimal criteria for each locus. The MCMC analysis lasted until the average standard deviation of split frequencies came below 0.01 with trees saved every 1000 generations. The first 25 % of saved trees were discarded as the ‘burn-in’ phase and posterior probabilities (PP) were determined from the remaining trees. The MP analysis was performed using PAUP v. 4.0b10 (Phylogenetic Analysis Using Parsimony; Swofford 2003). Phylogenetic relationships were estimated by heuristic searches with 1000 random addition sequences. Tree bisection-reconnection was used, with the branch swapping option set on ‘best trees’ only with all characters weighted equally and alignment gaps treated as fifth state. The tree length (TL), consistency index (CI), retention index (RI) and rescaled consistence index (RC) were calculated for the MP phylogenies and the bootstrap analysis (Hillis and Bull 1993) was based on 1000 replications. The ML analysis was performed under the GTR-GAMMA model of evolution using RAxML-VI-HPC v. 7.0.3 (Stamatakis 2006) with nonparametric bootstrapping using 1000 replicates. Trees were viewed in FigTree v. 1.1.2 (Rambaut 2009). The alignments used in the phylogenetic analyses, and resulting phylogenetic trees, were deposited in TreeBASE (submission ID S20001; https://treebase.org/).

## Results

### Morphology

Thirteen fungal isolates (Table 1) were obtained from yak rumen and faeces in Qinghai province, representing three morphological types. Nine of these isolates produced rhizoidal and monocentric thalli (Fig. 1a) with multi-flagellate zoospores (Fig. 1b) releasing through an irregularly ruptured apex of the mature sporangium, fitting with the morphology of *N. frontalis*. Two isolates possessed rhizoidal and polycentric thalli producing several sporangia terminally (Fig. 1c) with multi-flagellate zoospores (Fig. 1d), fitting with the morphology of *Orpinomyces joyonii*. The last two isolates possessed monocentric and bulbous thalli (Fig. 1e, g) with uni-flagellate zoospores (Fig. 1h) or bi-flagellate zoospores (Fig. 1f), fitting with the morphology of the genus *Caecomyces*.

### Phylogenetic analyses

Thirteen sequences of approximately 1500 bp long were generated for each of the isolates in this study, spanning from the end of the 18S nrRNA gene, the complete ITS1 and ITS2 region with intervening 5.8S nrRNA gene to the D1/D2 domain of the 28S nrRNA gene. The matrix statistics and related indices resulting from the phylogenetic analyses of the ITS1, the complete ITS and the partial LSU (D1/D2 domains) datasets are summarised in Table 2. Each of the three datasets covered all eight known genera, and included the ex-type sequences of *Oontomyces anksri* and *Buwchfawromyces eastonii*, as well as two common reference sequences in the *Neocallimastix* clade (isolates GE13 and SR4), two common reference sequences in the *Orpinomyces* clade (isolates OUS1 and KF2) and one in the *Anaeromyces* clade (isolate K9). None of our sequences fitted into any of the remaining clades published by Koetschan et al. (2014).

**Table 2:** A summary of matrix statistics for each alignment analysed phylogenetically in this study

Analysis	Loci analysed		
	ITS1	Complete ITS	D1/D2 domain of LSU
Statistics for the parsimony analyses			
Number of ingroup taxa	65	48	36
Number of nucleotide characters including gaps	398	871	759
Number of constant characters	9	104	429
Number of parsimony-informative characters	277	582	289
Number of parsimony-uninformative characters	112	185	41
Tree length	1629	2412	618
Consistency index (CI)	0.457	0.590	0.752
Retention index (RI)	0.781	0.796	0.885
Rescaled CI (RC)	0.370	0.469	0.666
Number of saved trees	1000	24	84
Statistics for the Bayesian analyses			
Substitution model	GTR + G	GTR + G	GTR + I + G
Number of generated trees	10,982	1922	1192
Number of trees discarded as the “burn-in” phase	2744	864	480
Number of trees used for final tree	8238	1442	894

#### ITS1 phylogeny

**Fig. 2:**
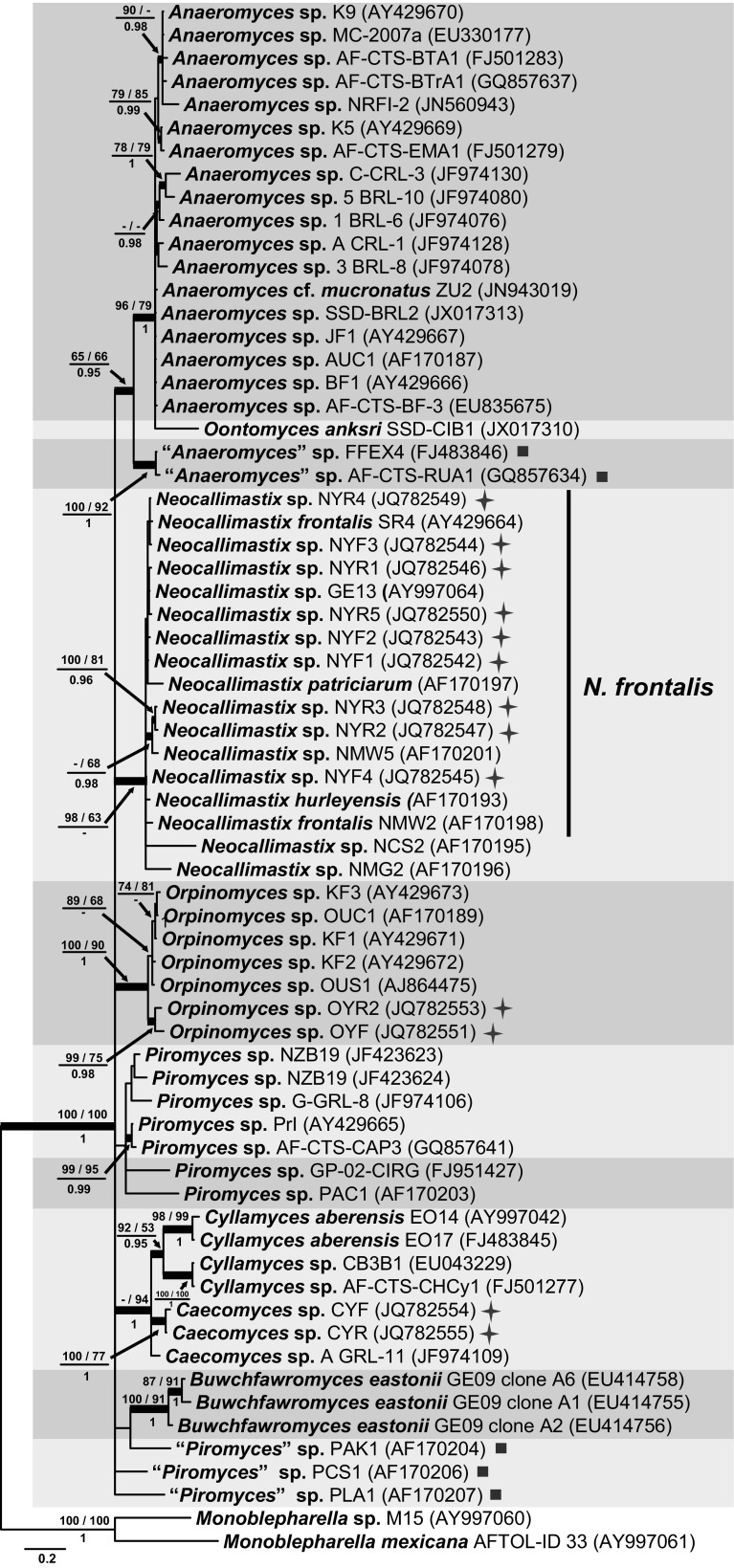
Consensus phylogram resulting from a Bayesian analysis of the ITS1 region, with the confidence values of bootstrap (BS) proportions from the MP analysis (before the *backslash*), the ML analysis (after the *backslash*) above branches, and the posterior probabilities (PP) from the Bayesian analysis below branches. The “-” indicates lacking statistical support (<50 % for ML-BS and MP-BS analyses; <0.90 for PP from Bayesian analyses). The branches with statistical support (MP-BS >50 %; ML-BS >50 %; PP >0.90) are highlighted with *thickened branches*. The tree is rooted to two isolates of *Monoblepharella*. Each genus clade is distinguished with *boxes* in different *colours* and lineages indicated with a *solid square* on the *right* represent isolates distinct from the eight known genera. Chinese isolates from yak are indicated with a star on the *right* side. The *scale bar* shows the expected number of changes per site

The BI consensus tree of ITS1 is presented in Fig. 2 with the respective PP, maximum parsimony bootstrap (MP-BS) and maximum likelihood bootstrap (ML-BS) support values indicated at the nodes. The ITS1 phylogenetic tree (Fig. 2) resolved only *Orpinomyces* (MP-BS = 100 %; ML-BS = 90 %; PP = 1.0), *Buwchfawromyces* (MP-BS = 100 %; ML-BS = 91 %; PP = 1.0), *Cyllamyces* (MP-BS = 92 %; ML-BS = 53 %; PP = 0.95) and *Neocallimastix* (MP-BS = 98 %; ML-BS = 63 %; PP < 0.95) as monophyletic lineages. In the *Neocallimastix* clade, the representatives of *N. patriciarum* and *N. hurleyensis* cluster together with isolate GE13 and 9 isolates from yak clustered closely with two representatives of *N. frontalis*, indicating that they represent the same species, *N. frontalis*. Two other isolates, NCS2 and NMG2, although on longer branches, also clustered in that clade. The genus *Anaeromyces* was divided into two sister lineages, one of which is designated here as the core *Anaeromyces* clade (MP-BS = 96 %; ML-BS = 79 %; PP = 1.0) and included the majority of the *Anaeromyces* isolates together with the isolate of *Oontomyces* on a long branch; the second *Anaeromyces* clade (MP-BS = 100 %; ML-BS = 92 %; PP = 1.0) contained two isolates: FFEX4 from cow faeces in UK (Griffith et al. 2009) and AF-CTS-RUA1 from rhinoceros faeces in India (Dagar et al. 2015). Koetschan et al. (2014) referred to this second clade as a distinct lineage “DT1”, which remains to be characterised morphologically as a novel genus. The two sister *Anaeromyces* clades had low statistic support (MP-BS = 65 %; ML-BS = 66 %; PP = 0.95) at their shared node. Two *Caecomyces* isolates from China formed a well-supported clade (MP-BS = 100 %; ML-BS = 77 %; PP = 1.0), but this clade did not include *Caecomyces* isolate A GRL-11. All three *Caecomyces* isolates clustered with the genus *Cyllamyces* (MP-BS < 50 %; ML-BS = 94 %; PP = 1.0). Isolates representing the genus *Piromyces* were polyphyletic and clustered at several positions throughout the phylogenetic tree. The backbone of the ITS1 phylogeny was collapsed to a basal polytomy and therefore no evidence was provided in the ITS1 tree to show the evolutionary relationships between any of the genera.

#### Complete ITS phylogeny

**Fig. 3:**
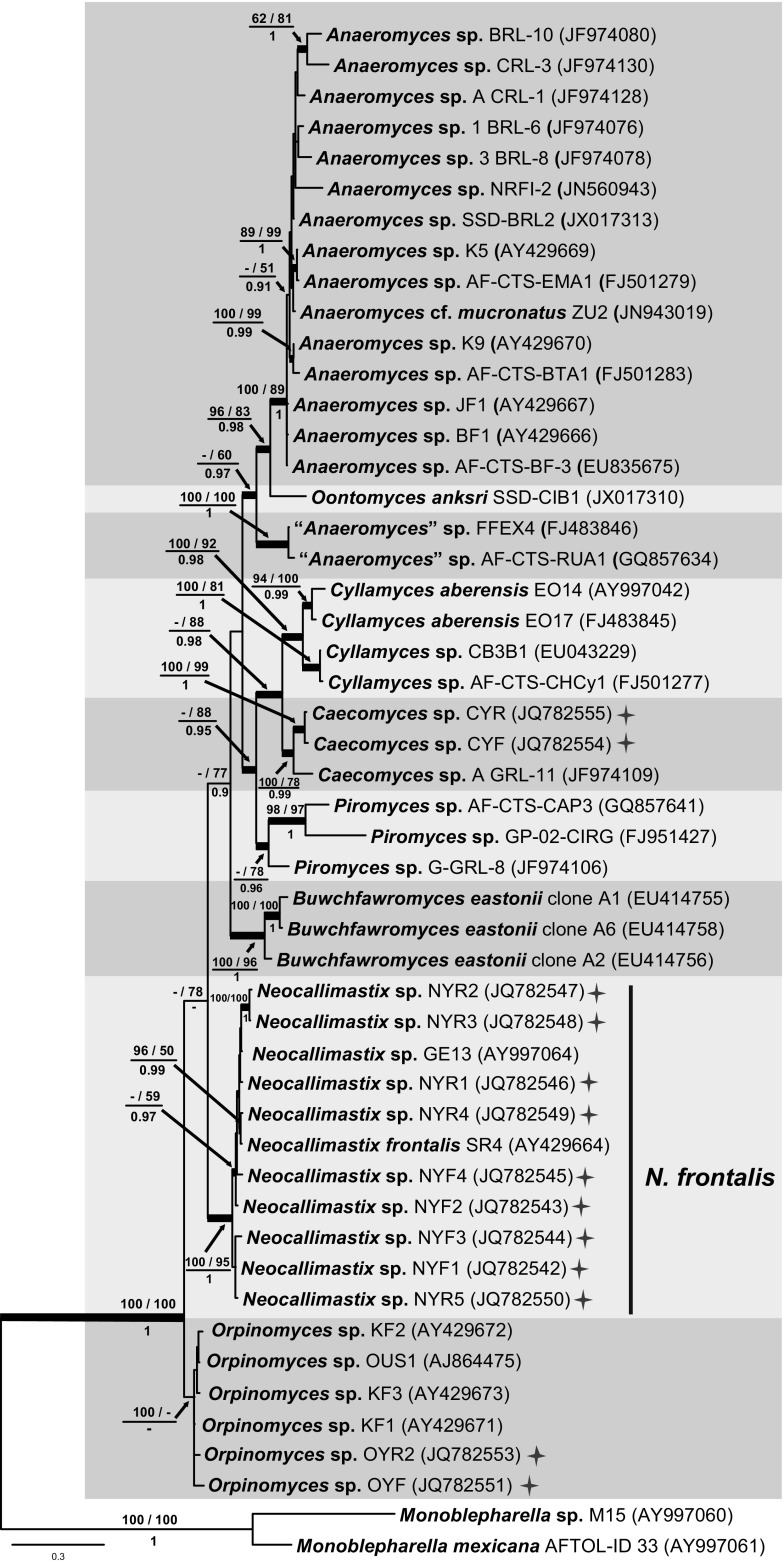
Consensus phylogram resulting from a Bayesian analysis of the complete ITS region, with the confidence values of bootstrap (BS) proportions from the MP analysis (before the *backslash*), the ML analysis (after the *backslash*) above branches, and the posterior probabilities (PP) from the Bayesian analysis below branches. The “-” indicates lacking statistical support (<50 % for ML-BS and MP-BS analyses; <0.90 for PP from Bayesian analyses). The branches with statistical support (MP-BS >50 %; ML-BS >50 %; PP >0.90) are highlighted with *thickened branches*. The tree is rooted to two isolates of *Monoblepharella*. Each genus clade is distinguished with boxes in different *colours*. Chinese isolates from yak are indicated with a star on the *right* side. The *scale bar* shows the expected number of changes per site

The BI consensus tree of the complete ITS is presented in Fig. 3 with the respective PP, MP-BS and ML-BS values indicated at the nodes. With the addition of phylogenetic information from ITS2, the complete ITS tree (Fig. 3) confirmed the monophyly of *Cyllamyces* (MP-BS = 100 %; ML-BS = 92 %; PP = 0.98), *Buwchfawromyces* (MP-BS = 100 %; ML-BS = 96 %; PP = 1.0) and *Neocallimastix* (MP-BS = 100 %; ML-BS = 95 %; PP = 1.0), and also resolved *Caecomyces* (MP-BS = 100 %; ML-BS = 78 %; PP = 0.99) as a monophyletic lineage. The highly supported *Neocallimastix* clade (MP-BS = 100 %; ML-BS = 95 %; PP = 1.0) included nine isolates from yak and the reference isolate (SR4) of *N. frontalis*, providing further evidence that our *Neocallimastix* isolates belong to the species *N. frontalis*. Similar to the ITS1 phylogeny, the genus *Anaeromyces* was again divided into two clades, with *Oontomyces* represented by a single lineage intermediate between the two clades. The node joining all three clades was poorly supported (MP-BS <50 %; ML-BS = 60 %; PP = 0.97), whereas the core clade (MP-BS = 100 %; ML-BS = 89 %; PP = 1) and smaller clade (MP-BS = 100 %; ML-BS = 100 %; PP = 1) containing isolates identified morphologically as *Anaeromyces* were both well-supported. *O. anksri* was placed basal to the core *Anaeromyces* clade with statistical support (MP-BS = 96 %; ML-BS = 83 %; PP = 0.98). With limited sampling due to lack of full-length sequences, only three *Piromyces* isolates could be included in this analysis and these clustered together with low support (MP-BS < 50 %; ML-BS = 78 %; PP = 0.96) and showed a close relationship (MP-BS < 50 %; ML-BS = 88 %; PP = 0.95) to the *Cyllamyces*/*Caecomyces* clade. The *Cyllamyces* clade (MP-BS = 100 %; ML-BS = 92 %; PP = 0.98) and *Caecomyces* clade (MP-BS = 100 %; ML-BS = 78 %; PP = 0.99) were both well-supported and cluster as sister clades with variable support (MP-BS < 50; ML-BS = 88 %; PP = 0.98), but no species of *Caecomyces* identified to species level were available. The *Orpinomyces* clade in the complete ITS phylogeny was only fully supported in the maximum parsimony analysis (MP-BS = 100 %; ML-BS < 50 %; PP < 0.90) but no species identified to species level were available.

#### LSU phylogeny

**Fig. 4:**
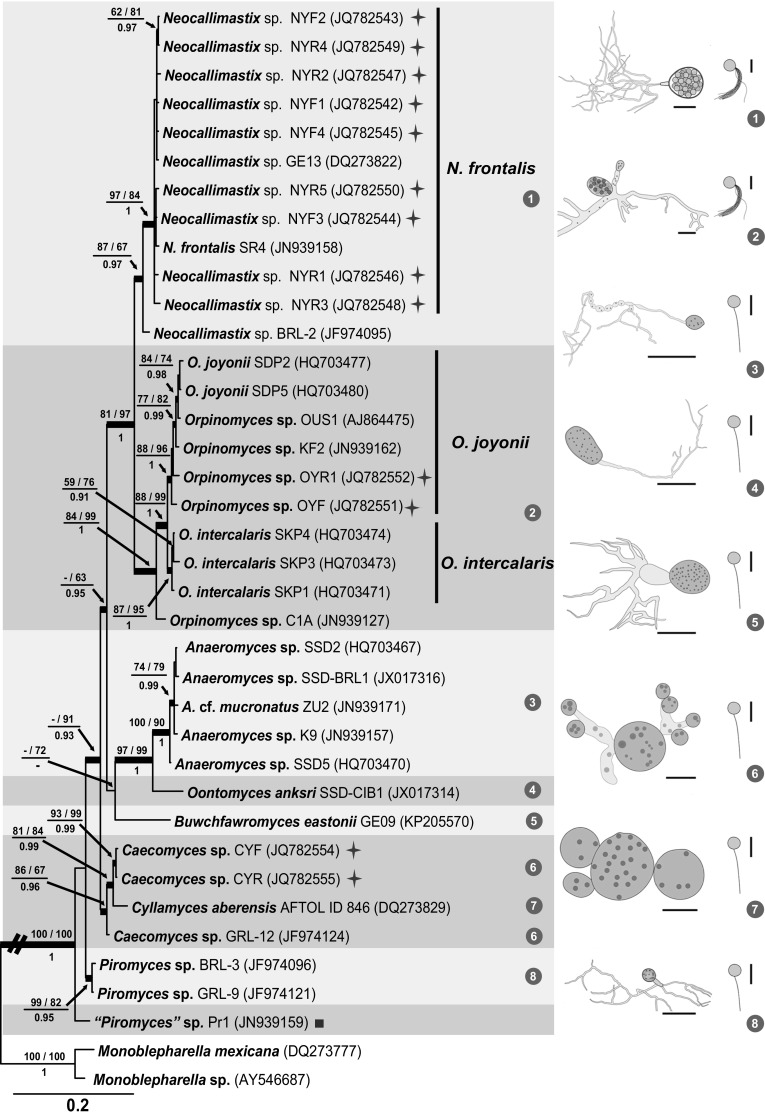
Consensus phylogram resulting from a Bayesian analysis of the D1/D2 domain of LSU, with the confidence values of bootstrap (BS) proportions from the MP analysis (before the *backslash*), the ML analysis (after the *backslash*) above branches, and the posterior probabilities (PP) from the Bayesian analysis below branches. The “-” indicates lacking statistical support (<50 % for ML-BS and MP-BS analyses; <0.90 for PP from Bayesian analyses). The branches with statistical support (MP-BS >50 %; ML-BS >50 %; PP >0.90) are highlighted with* thickened branches*. The tree is rooted to two isolates of *Monoblepharella*. Each genus clade is distinguished with* boxes* in different *colours* and the lineage indicated with a *solid square* on the *right* represents an isolate distinct from the eight known genera. Chinese isolates from yak are indicated with a star on the *right*. The *scale bar* on the phylogeny shows the expected number of changes per site. Morphological characteristics of each of the known genera are illustrated to the *right* of the tree. The illustrations of the zoospores and the thalli of *Neocallimastix* and *Orpinomyces* were derived from this study (Fig. 1d h, a, c, respectively); the thallus of *Anaeromyces* was derived from Fliegerová et al. (2004); the thallus of *Oontomyces* was derived from Dagar et al. (2015); the thallus of *Buwchfawromyces* was derived from Callaghan et al. (2015); the thallus of *Cyllamyces* was derived from Ozkose et al. (2001); and the thalli of *Caecomyces* and *Piromyces* were derived from Gruninger et al. (2014). Thalli are shown on the *left* and zoospores are on the *right* (*scale bars* zoospores = 10 µm; sporangium 1, 2, 7 = 20 µm; 3 = 100 µm; 4, 5, 6, 8 = 50 µm). Thalli of *Neocallimastix* (1) and *Orpinomyces* (2) are illustrated with a sporangium filled with zoospores. Thalli of the other genera are illustrated with nuclei as *black dots* inside the sporangia or mycelia, for example those in *Anaeromyces* (3)

The BI consensus tree of LSU is presented (Fig. 4) with the respective PP, MP-BS and ML-BS values indicated at the nodes. In the LSU phylogeny (Fig. 4), the monophyletic clades of *Neocallimastix* (MP-BS = 87 %; ML-BS = 67 %; PP = 0.97) and *Orpinomyces* (MP-BS = 84 %; ML-BS = 99 %; PP = 1) cluster as sister lineages (MP-BS = 81 %; ML-BS = 97 %; PP = 1). The reference isolate (SR4) of *N. frontalis*, and isolates GE13 and 9 from yak again clustered in a well-supported clade (MP-BS = 97 %; ML-BS = 84 %; PP = 1). Two yak *Orpinomyces* isolates grouped with the representative isolates of *O. joyonii* (MP-BS = 88 %; ML-BS = 96 %; PP = 1), a close sister to the *O. intercalaris* clade (MP-BS = 87 %; ML-BS = 95 %; PP = 1). *O. anksri* clustered again basal to the core *Anaeromyces* with high support (MP-BS = 97 %; ML-BS = 99 %; PP = 1); the core *Anaeromyces* clade is well-supported (MP-BS = 100 %; ML-BS = 90 %; PP = 1). No LSU data were available for the second smaller *Anaeromyces* clade present in both ITS trees. The position of *Buwchfawromyces* was poorly supported in the LSU phylogeny (MP-BS < 50 %; ML-BS = 72 %; PP < 0.9). The two Chinese *Caecomyces* isolates clustered together with high support (MP-BS = 93 %; ML-BS = 99 %; PP = 0.99) in a clade containing *C. aberensis* and *Caecomyces* isolate GRL-12 (MP-BS = 86 %; ML-BS = 67 %; PP = 0.96). Similar to the ITS1 and complete ITS phylogenies, *Piromyces* was again polyphyletic. Two of the *Piromyces* isolates (BRL3 and GRL9) formed a clade (MP-BS = 99 %; ML-BS = 82 %; PP = 0.95 %), but the third *Piromyces* isolate (Pr1) clustered basal to all other species included in the phylogeny. The morphologies of the eight genera are illustrated to the right of the LSU tree to match the core morphological features of the genera with their phylogenetic relationships (Fig. 4).

## Discussion

Yak (*Bos grunniens*) exhibits unique adaptations to its fibre-rich diet, representing a promising reservoir of enzymes for degrading plant biomass. Over 95 % of yak populations exist in China, but only limited information is available about the composition of its anaerobic fungal population (Feng 2005; Wei et al. 2016). Our morphological investigation and phylogenetic analyses show the presence of a *Neocallimastix* species (*N. frontalis*) and an *Orpinomyces* species (*O. joyonii*), and also showed the existence of the bulbous-type genus *Caecomyces* in yak for the first time.

Unlike all the other members in the kingdom Fungi, their strict anaerobic nature and strict temperature requirement make it extremely difficult to retain anaerobic fungi in a viable and healthy state for study. The lack of a reliable method for long-term preservation of pure cultures of anaerobic fungi (Gruninger et al. 2014; Haitjema et al. 2014) resulted in the loss of the cultures of most described species in *Neocallimastigomycota*. The discovery of high variation in morphology further challenged the taxonomic study of *Neocallimastigomycota* (Barr et al. 1989; Ho and Barr 1995). Application of molecular technique has provided a route towards a more robust reappraisal of taxa in this group of fungi (Gruninger et al. 2014). As noted in the Introduction, genera of anaerobic fungi (Ho and Barr 1995; Ozkose et al. 2001; Gruninger et al. 2014) were traditionally defined by the thallus morphology (rhizoidal/bulbous and polycentric/monocentric) and the number of flagella per zoospore (uni-flagellate/multi-flagellate). A reappraisal of the morphologically-defined generic concept based on the phylogenetic analyses of three loci in this study (Figs. 2, 3, 4) shows that when two taxa have different combinations of thallus and zoospore morphology they indeed belong to different genera, while also showing that taxa with the same morphological combination can in fact belong to different genera. For example, *Oontomyces*, *Buwchfawromyces* and isolates that are morphologically *Piromyces*-like but phylogenetically distinct from *Piromyces* share the same morphological combination (rhizoidal and monocentric thalli with uni-flagellate zoospores) but these four groups are phylogenetically not congeneric. Isolates FFEX4 and AF-CTS-RUA1 share the same morphological combination (rhizoidal and monocentric thalli with uni-flagellate zoospores) with the core *Anaeromyces* clade, but based on the molecular data should represent a separate genus (“DT1”, Koetschan et al. 2014). Unfortunately, we did not have access to LSU sequences of these isolates to determine their position in the LSU phylogeny. Species identification is quite problematic because of the difficulty in comparative investigation of all samples using consistent criteria, with the exception of the genera *Neocallimastix* and *Orpinomyces*. After the monographic study of Ho and Barr (1995), only *O. joyonii* and *O. intercalaris* were recognised in the genus *Orpinomyces*; and only *N. frontalis* and *N. hurleyensis* in the genus *Neocallimastix*. The D1/D2 domain of LSU was reported to be capable of distinguishing the two species of *Orpinomyces* based on the analysis of a dataset without representatives of any other genera (Dagar et al. 2011). Our LSU analyses supported the distinction between *O. joyonii* and *O. intercalaris*, although they seemed genetically very close to each other (Fig. 4). Phylogenetic analyses of three loci in this study (Figs. 2, 3, 4) indicated that *N. hurleyensis* should be a synonym of *N. frontalis*, which implies that *N. frontalis* is the only species successfully isolated and cultured in the genus *Neocallimastix*. On the other hand, recent evidence from cultivation-independent surveys and sequence analyses suggest the presence of several novel lineages, as well as a much higher diversity of taxa in each of the known genus clades, including the *Neocallimastix* clade and the *Orpinomyces* clade (Nicholson et al. 2010; Liggenstoffer et al. 2010; Koetschan et al. 2014), implying that potential candidates of novel species and genera exist. The development of advanced culturing techniques is thus encouraged in order to attempt to isolate representatives of these uncharacterised taxa (Gruninger et al. 2014).

A polycentric growth habit was once believed to be of significance in the evolution of zoosporic fungi, representing a potential route for the evolution of more advanced fungal forms (Barr 1983; Ozkose et al. 2001). Gruninger et al. (2014) suggested that *Neocallimastix* was closely related with *Orpinomyces*, both genera having polyflagellate zoospores. In our analyses, both ITS1 and the complete ITS phylogenies failed to provide credible support for the relationship of *Neocallimastix* and *Orpinomyces*, but they were strongly supported to be sister lineages in our LSU phylogeny. This study also supports the belief that *Caecomyces* and *Cyllamyces* are closely related, sharing bulbous thalli (Gruninger et al. 2014). *Caecomyces* is generally defined by having monocentric thalli compared to *Cyllamyces* defined by having polycentric thalli (Gruninger et al. 2014). However, the morphological variability of the genus *Caecomyces* confused the delimitation of these two bulbous genera. It has been observed that isolates of *Caecomyces* produced several bulbous holdfasts and several sporangia, especially in old (more than 20 h) cultures (Wubah et al. 1991a; Ho and Barr 1995), and even exhibited multisporangiate thalli with sporangia sympodially arising from unbranched sporangiophores (Chen et al. 2007). In this study, all of the analyses (except for the parsimony bootstrap analyses of ITS1 and the complete ITS), supported a shared ancestry for *Caecomyces* and *Cyllamyces*. The ITS1 phylogeny did not resolve all isolates of *Caecomyces* as monophyletic. This could be explained by a lack of resolution when ITS1 alone is used to delimit these two genera. Although the LSU phylogeny failed to resolve the two genera, it is quite possible that sampling could be playing a role since only one LSU sequence of *Cyllamyces* was available to be included. The complete ITS sequences, which represent a wider sampling than LSU, suggest that *Caecomyces* and *Cyllamyces* could be distinct genera. More LSU sequences representing these two genera are needed to reach a final conclusion about whether the difference between *Cyllamyces* and *Caecomyces* should be at genus level or at species level. The complete ITS and LSU phylogenies supported *Oontomyces* as a sister lineage to *Anaeromyces*, whereas the phylogenetic position of *Buwchfawromyces* could not be consistently fixed by all analyses and alignments. In all analyses and alignments, isolates of *Piromyces* were polyphyletic, indicating that this genus requires a more detailed revision utilising longer sequences from more cultures and critical morphological examination of their microscopic characteristics. Care should therefore be taken to assign next-generation sequencing data from environmental samples to this genus.

Our phylogenetic analyses based on different loci allow us to evaluate the phylogenetic value of the complete ITS, the ITS1 region, and the D1/D2 region of LSU. The complete ITS region has been designated as official barcode for fungi (Schoch et al. 2012). Since Brookman et al. (2000) proposed the use of ITS1 for the classification of the gut fungi, it has become the most widely used amplicon for the study of taxonomy and community composition of *Neocallimastigomycota*. However, the highly variable nature of the ITS1 makes it difficult to align obtained sequences to get a reliable phylogenetic tree. In order to solve this issue, an ITS1 secondary structure prediction approach was proposed by Koetschan et al. (2014). Their revised ITS1 phylogenetic tree (Fig. 4 in Koetschan et al. 2014) showed better relationships between the known genera as well as several uncultured lineages, which is similar to the phylogenetic relationships revealed in the complete ITS tree here (Fig. 3) to some extent. For example, the genus *Orpinomyces* was located at the basis of the whole tree rather than forming a sister to *Neocallimastix*, and the *Piromyces* clade was close to the *Cyllamyces*/*Caecomyces* clade. On the other hand, intragenomic variation within the ITS region has been noted which would cause problems for direct sequencing PCR products (Li and Heath 1992; Brookman et al. 2000). A recent study has shown significant differences between ITS1 sequences obtained from different transformed colonies after cloning of a PCR amplicon of one isolate (GE09) of *Buwchfawromyces eastonii*. This recently described species was isolated from buffalo faeces in west Wales, but the ITS1 phylogeny (Fig. 3 in Callaghan et al. 2015) showed that clone A2 (GenBank EU414756) and clone A1 (GenBank EU414755) were more distant to clone A6 (EU414758) than to several uncultured sequences obtained from red deer in New Zealand. For this reason, further study is strongly needed to clarify how high the intragenomic variation in the ITS region of isolates in different genera is before utilising ITS1 in future studies classifying and identifying anaerobic fungi. On the other hand, no intragenomic variation was shown within the D1/D2 region of LSU in the study of *B. eastonii* (Fig. 2 in Callaghan et al. 2015). Furthermore, the LSU data obtained in this study included only a few gaps after sequence alignment using MAFFT v.7 (Katoh and Standley 2013); therefore the phylogeny based on this region is less sensitive to artefacts introduced by the sequence alignment. Except for *Cyllamyces* and *Caecomyces*, the LSU alignment provided a better resolved and supported phylogeny of the different genera.

Based on the LSU phylogeny, a hypothetical evolution can be proposed here that a *Piromyces*-like organism with monocentric thalli and uniflagellate zoospores might have been the ancestral lineage, from which members of the *Cyllamyces*/*Caecomyces* clade (with bulbous thalli and uniflagellate zoospores) were derived first, followed by *Anaeromyces* (with uniflagellate zoospores but polycentric thalli) and then *Neocallimastix*/*Orpinomyces* (with polyflagellate zoospores). The morphologically *Piromyces*-like genera *Oontomyces* and *Buwchfawromyces* are basal to the *Anaeromyces* clade, implying that the evolutionary process associated with the combination of monocentric thalli and uniflagellate zoospores happened more than once in the order.

With the great progress in both morphological and molecular study of anaerobic fungi, it is a good time now to establish a stable and reliable classification system in *Neocallimastigomycota*. It is necessary to recollect new isolates for all the known species for which type cultures have not been available. Form these fresh isolates, more inclusive morphological data and sequence data could be generated promptly by critical examination of morphology under light microscope together with sequencing of multiple DNA loci before the isolates are threatened by the lack of a reliable method for their long-term preservation. These data will be used to epi- or neotypify all the old names of the known species without type cultures. The release of genomic data could provide suitable protein-coding genes to supplement nrDNA sequence data, which would help to further resolve the phylogenetic relations between each of the known genera, and to more accurately delimit species within each genus, paving the way to the discovery of new taxa.

## Conclusions

This study presents the first summary of the current molecular knowledge of rumen fungi of *Neocallimastigomycota* known from culture. The phylogenetic informativity of the ITS1 region, which is commonly used in DNA analyses of this group of fungi, was compared to phylogenies derived from the complete ITS region as well as the D1/D2 of the LSU region. Our data show that ITS1, or the complete ITS, alone do have limitations in the generic phylogeny and classification of rumen fungi, for example for *Anaeromyces*/*Oontomyces* in the ITS1 tree and for *Orpinomyces* in the ITS tree. However, in general the use of LSU does provide molecular support for most of the currently accepted genera of rumen fungi, and it does resolve the relationships between the genera much clearer. The only exception being members of *Piromyces*, which we hypothesise based on the presented LSU phylogeny to represent the ancestral morphology of the rumen fungi. This could also explain why this morphology occurs at multiple positions in the phylogeny; for some of those occurrences, novel genera with a *Piromyces*-like morphology were introduced in recent years, e.g. *Oontomyces* and *Buwchfawromyces*. To further stabilise the phylogeny of rumen fungi, a multi-locus approach may be useful. By both morphology and molecular phylogeny, the 13 isolates obtained from yak faeces and rumen digesta in China could be assigned to *N. frontalis* (9 isolates), *O. joyonii* (2 isolates) and an unclassified species of *Caecomyces* (2 isolates), giving more insight into the anaerobic fungal diversity in yak. Our *Caecomyces* isolates were genetically different from other taxa published in this genus with molecular evidence. Since all the described species of *Caecomyces* lack sequence data from their ex-type or authentic cultures, no new taxa were proposed in this study.

## Electronic supplementary material

Below is the link to the electronic supplementary material.

Supplementary material 1 (DOCX 29 kb)
