# Supplementary material for: Phylogeny of anaerobic fungi (phylum Neocallimastigomycota), with contributions from yak in China
Source: Antonie Van Leeuwenhoek. 2016 Oct 12;110(1):87–103. doi: 10.1007/s10482-016-0779-1 (PMC5222902; doi:10.1007/s10482-016-0779-1)
Supplement: Supplementary file 1 — Supplementary material 1 (DOCX 29 kb) [file 10482_2016_779_MOESM1_ESM.docx]

**Table S1.** Information on the reference sequences included in the phylogenetic analyses in this study

| Taxon | Strain | Source | GenBank accession numbers | | Reference |
| --- | --- | --- | --- | --- | --- |
|  |  |  | ITS1 / ITS | LSU |  |
| *Anaeromyces* cf. *mucronatus* | ZU2 | － | JN943019 | JN939171 | Schoch et al. (2012) |
| *Anaeromyces* sp. | 1 BRL-6 | Buffalo rumen, India | JF974076 | － | Dagar et al. (2015) |
| *Anaeromyces* sp. | 3 BRL-8 | Buffalo rumen, India | JF974078 | － | Dagar et al. (2015) |
| *Anaeromyces* sp. | 5 BRL-10 | Buffalo rumen, India | JF974080 | － | Dagar et al. (2015) |
| *Anaeromyces* sp. | A CRL-1 | Cattle rumen, India | JF974128 | － | Dagar et al. (2015) |
| *Anaeromyces* sp. | AF-CTS-BF-3 | Buffalo faeces | EU835675 | － | Dagar et al. (2015) |
| *Anaeromyces* sp. | AF-CTS-BTrA1 | Faeces of nilghai, India | GQ857637 | － | Callaghan et al. (2015) |
| *Anaeromyces* sp. | AF-CTS-EMA1 | － | FJ501279 | － | Dagar et al. (2015) |
| *Anaeromyces* sp. | AF-CTS-RUA1 | Rhinoceros faeces, India | GQ857634 | － | Dagar et al. (2015) |
| *Anaeromyces* sp. | BF1 | Faeces of American bison, Prague, Czech Republic | AY429666 | － | Fliegerová et al. (2004) |
| *Anaeromyces* sp. | C-CRL-3 | Cattle rumen, India | JF974130 | － | Dagar et al. (2015) |
| *Anaeromyces* sp. | FFEX4 | Cow faeces, UK | FJ483846 | － | Griffith et al. (2009) |
| *Anaeromyces* sp. | AUC1 | Cow, UK | AF170187 | － | Brookman et al. (2000) |
| *Anaeromyces* sp. | JF1 | Deer faeces, Prague, Czech Republic | AY429667 | － | Fliegerová et al. (2004) |
| *Anaeromyces* sp. | K5 | Cow rumen, Prague, Czech Republic | AY429669 |  | Fliegerová et al. (2004) |
| *Anaeromyces* sp. | K9 | Cow rumen, Prague, Czech Republic | AY429670 | JN939157 | Fliegerová et al. (2004) |
| *Anaeromyces* sp. | MC-2007a | Goat, India | EU330177 | － | Callaghan et al. (2015) |
| *Anaeromyces* sp. | NRFI-2 | Cattle faeces, Karnal, India | JN560943 | － | Sirohi et al. (2013) |
| *Anaeromyces* sp. | NRFI-2 | Cattle faeces, Karnal, India | JN560943 | － | Sirohi et al. (2013a) |
| *Anaeromyces* sp. | SSD-BRL2 | Ruminant, India | JX017313 | JX017317 | Dagar et al. (2015) |
| *Anaeromyces* sp. | SSD2 | Buffalo rumen, India | － | HQ703467 | Dagar et al. (2015) |
| *Anaeromyces* sp. | SSD5 | Buffalo rumen, India | － | HQ703470 | Dagar et al. (2015) |
| *Buwchfawromyces eastonii* | GE09 | Buffalo faeces, UK | EU414756 | KP205570 | Callaghan et al. (2015) |
| *Caecomyces* sp. | A GRL-11 | Goat, India | JF974109 | － | Callaghan et al. (2015) |
| *Caecomyces* sp. | GRL-12 | Goat, India | － | JF974124 | Dagar et al. (2015) |
| *Cyllamyces aberensis* | EO14 (AFTOL-ID 846) | － | AY997042 | DQ273829 | James et al. (2006) |
| *Cyllamyces aberensis* | EO17 | Cow faeces, UK | FJ483845 | － | Griffith et al. (2009) |
| *Cyllamyces* sp. | AF-CTS-CHCy1 | － | FJ501277 | － | Dagar et al. (2015) |
| *Cyllamyces* sp. | CB3B1 | － | EU043229 | － | Dagar et al. (2015) |
| *Monoblepharella mexicana* | BK 78-1 (AFTOL-ID 33 | － | AY997061 | DQ273777 | James et al. (2006) |
| *Monoblepharella* sp. | M15 (AFTOL-ID 25) | － | AY997060 | AY546687 | James et al. (2006) |
| *Neocallimastix frontalis* | NMG2 | Goat, Malaysia | AF170196 | － | Brookman et al. (2000) |
| *Neocallimastix frontalis* | NMW2 | Water buffalo, Malaysia | AF170198 | － | Brookman et al. (2000) |
| *Neocallimastix frontalis* | SR4 | Cow rumen, Prague, Czech Republic | AY429664 | JN939158 | Fliegerová et al. (2004) |
| *Neocallimastix hurleyensis* | － | Sheep, UK | AF170193 | － | Brookman et al. (2000) |
| *Neocallimastix patriciarum* | NMW1 | Water buffalo, Malaysia | AF170197 | － | Brookman et al. (2000) |
| *Neocallimastix* sp. | 2 BRL-2 | Buffalo, India | － | JF974095 | Dagar et al. (2015) |
| *Neocallimastix* sp. | GE13 (AFTOL-ID 638) | － | AY997064 | DQ273822 | James et al. (2006) |
| *Neocallimastix* sp. | NCS2 | Sheep, China | AF170195 | － | Brookman et al. (2000) |
| *Neocallimastix* sp. | NMW5 | Water buffalo, Malaysia | AF170201 | － | Brookman et al. (2000) |
| *Oontomyces anksri* | SSD-CIB1 | Digestive tract of camel, India | JX017310 | JX017314 | Dagar et al. (2015) |
| *Orpinomyces intercalaris* | SKP1 | Buffalo rumen, India | － | HQ703471 | Dagar et al. (2011) |
| *Orpinomyces intercalaris* | SKP3 | Buffalo rumen, India | － | HQ703473 | Dagar et al. (2011) |
| *Orpinomyces intercalaris* | SKP4 | Buffalo rumen, India | － | HQ703474 | Dagar et al. (2011) |
| *Orpinomyces joyonii* | SDP2 | Buffalo rumen, India | － | HQ703477 | Dagar et al. (2011) |
| *Orpinomyces joyonii* | SDP5 | Buffalo rumen, India | － | HQ703480 | Dagar et al. (2011) |
| *Orpinomyces* sp. | C1A | － | － | JN939127 | Schoch et al. (2012) |
| *Orpinomyces* sp. | KF1 | Cow rumen, Prague, Czech | AY429671 | － | Fliegerová et al. (2004) |
| *Orpinomyces sp.* | KF2 | Cow rumen, Prague, Czech | AY429672 | JN939162 | Fliegerová et al. (2004) |
| *Orpinomyces* sp. | KF3 | Cow rumen, Prague, Czech | AY429673 | － | Fliegerová et al. (2004) |
| *Orpinomyces* sp. | OUC1 | Cow, UK | AF170189 | － | Brookman et al. (2000) |
| *Orpinomyces sp.* | OUS1 | Sheep, UK | AJ864475 | AJ864475 | Nicholson et al. (2005) |
| *Piromyces* sp. | AF-CTS-CAP3 | Animal faeces, India | GQ857641 | － | Callaghan et al. (2015) |
| *Piromyces* sp. | BRL-3 | Buffalo, India | － | JF974096 | Dagar et al. (2015) |
| *Piromyces* sp. | G-GRL-8 | Goat, India | JF974106 | － | Dagar et al. (2015) |
| *Piromyces* sp. | GP-02-CIRG | Goat rumen, India | FJ951427 | － | Callaghan et al. (2015) |
| *Piromyces* sp. | GRL-9 | Goat, India | － | JF974121 | Dagar et al. (2015) |
| *Piromyces* sp. | NZB19-01 | Cow faeces, New Zealand | JF423623 | － | Kittelmannet al. (2012) |
| *Piromyces* sp. | NZB19-02 | Cow faeces, New Zealand | JF423624 | － | Kittelmannet al. (2012) |
| *Piromyces* sp. | PAC1 | Cow, Australia | AF170203 | － | Brookman et al. (2000) |
| *Piromyces* sp. | PAK1 | Kangaroo, Australia | AF170204 | － | Brookman et al. (2000) |
| *Piromyces* sp. | PCS1 | Sheep, China | AF170206 | － | Brookman et al. (2000) |
| *Piromyces* sp. | PLA1 | Alpaca, Chile | AF170207 | － | Brookman et al. (2000) |
| *Piromyces* sp. | PrI | －, Czech Republic | AY429665 |  | Fliegerová et al. (2004), |
| *Piromyces* sp. | Pr1 | －, Czech Republic |  | JN939159 | Dagar et al. (2015) |
